# The p38/MK2 Axis in Monocytes of Fibromyalgia Syndrome Patients: An Explorative Study

**DOI:** 10.3390/medicina57040396

**Published:** 2021-04-19

**Authors:** Boya Nugraha, Renate Scheibe, Christoph Korallus, Matthias Gaestel, Christoph Gutenbrunner

**Affiliations:** 1 Department of Rehabilitation Medicine, Hannover Medical School, Carl-Neuberg-Str.1, 30625 Hannover, Germany; Korallus.Christoph@mh-hannover.de; 2 Institute of Cell Biochemistry, Hannover Medical School, Carl-Neuberg-Str.1, 30625 Hannover, Germany; Scheibe.Renate@mh-hannover.de (R.S.); Gaestel.Matthias@mh-hannover.de (M.G.)

**Keywords:** fibromyalgia, pain, p38, MAPK, depression, mood

## Abstract

*Background and Objectives*: The aetiology and pathomechanism of fibromyalgia syndrome 12 (FMS) as one of chronic pain syndromes still need to be further elucidated. Mitogen-activated protein kinase (MAPK) pathway has been proposed as a novel approach in pain management. Since the major symptom of fibromyalgia syndrome (FMS) patients is pain, it became of interest whether MAPK pathways, such as the stress-activated p38 MAPK/MK2 axis, are activated in FMS patients. Therefore, this study aimed at determining p38 MAPK/MK2 in FMS patients. *Materials and Methods*: Phosphorylation of MAPK-activated protein kinases 2 (MK2), a direct target of p38 MAPK, was measured in monocytes of FMS and healthy controls (HCs) to monitor the activity of this pathway. *Results*: The mean level of phosphorylated MK2 was fivefold higher in FMS patients as compared to HCs (*p* < 0.001). Subgroup analysis revealed that antidepressants did not influence the activity of MK2 in FMS patients. *Conclusions*: This result indicates that the p38/MK2 pathway could be involved in the pathomechanism of FMS, could act as a clinical marker for FMS, and could be a possible target for pain management in FMS patients.

## 1. Introduction

The aetiology and pathomechanism of fibromyalgia syndrome (FMS) are still remaining elusive. Besides the alteration of pain sensitization in the peripheral and central nervous system [1,2,3], one of the most proposed hypotheses in initiation of FMS is the aberration of the hypothalamus-pituitary-adrenal (HPA) axis [4], which leads to dysregulation of glucocorticoid levels in FMS patients [5]. This result can also alter immune systems, as innate and adaptive immune systems are influenced by glucocorticoids [6]. It has been hypothesized that FMS may be related to processes associated with chronic stress [4]. For example, depression as a long-term consequence of stress can enhance glucocorticoid receptor (GR) function in an animal model [7]. Furthermore, chronic stress is known to downregulate the secretion of dopamine leading to hypersensitivity to pain (hyperalgesia) [8]. Thus, both psychologic stressors and inflammation may be relevant in initiation of FMS because of synergistic inputs on cytokine-mediated responses from the dopamine system in nucleus accumbens [9,10].

The mitogen-activated protein kinases (MAPKs) are a family of serine/threonine-specific kinases. The family consists of three major members in mammals: extracellular signal-regulated kinases (ERK, including ERK1/2), c-Jun N-terminal kinases (JNK, including JNK1, JNK2, and JNK3), and p38 (including p38α, p38ß, p38γ, and p38δ), which represent three separate signaling pathways. The primary extracellular stimulators of the p38 MAPK signaling pathway in various cell types are stress and cytokines [11]. Notably, the p38 MAPK pathway is not only activated by cytokines, but in turn, also controls pro-inflammatory cytokine production, such as interleukin-6 (IL-6) and tumor necrosis factor (TNF)-α [12]. In addition, the p38 MAPK pathway is activated under inflammatory and neuropathic pain conditions, and activated kinases of this pathway can be used as molecular and cellular markers for pain conditions [13]. Inhibition of p38 MAPK is effective to reduce rheumatoid arthritis activity and inflammatory pain [14,15]. The p38 MAPK inhibitor dilmapimod significantly reduced neuropathic pain [16].

Monocytes represent 10% and 4% of leukocytes in human and mouse blood, respectively [17]. Monocytes can further differentiate into tissue macrophages, dendritic cells and osteoclasts, which are influenced by different types of cytokines [18,19]. They are commonly defined by cell surface expression of CD11b and CD14 in humans [20]. Monocytes are one main source of pro-inflammatory cytokine production, such as IL-1ß, IL-6, and TNF-α, in response to pathogenic infection [21]. As the p38 kinase was originally identified as a MAPK activated by lipoplysaccharide (LPS) stimulation of monocytes [22] and MK2 determines the posttranscriptional regulation of cytokine biosynthesis [23], monocytes are of particular interest for studying p38 MAPK signaling.

The journey to elucidate the aetiology and pathomechanisms of FMS patients has not been coming to the end, yet. So far, no implication of the MAPK signaling cascade in the development of FMS-associated symptoms has been reported. The determination of the MAPK signaling in this group of patients could gain knowledge in this field. To investigate whether activation of the p38 MAPK-MK2 pathway in monocytes could act as a critical mediator for FMS patients, our approach was to compare phosphorylation of p38 MAPK substrate, MK2, in blood monocytes of FMS patients and healthy subjects. Our hypothesis was that the level of MK2 might be altered in FMS patients because of its stress-dependent activation and its role in pain-related mechanisms.

A rapid method has recently been developed for separating mouse monocytes from other blood cells for detecting expression and activation levels of the stimulated p38 MAPK-MK2 pathway [24]. In addition, the inhibition of LPS-induced TNF-α production in whole blood by specific p38 MAPK kinase inhibitors correlated with the inhibition of MK2 phosphorylation in blood monocyte [24], suggesting that the blood monocyte-based assay can be a valuable approach to determine p38 MAPK-MK2 function. However, this method had to be adapted to human samples.

## 2. Materials and Methods

To answer the question, we designed a cross-sectional study comparing FMS patients with healthy controls (HCs). All procedures were carried out with the written informed consent of the subjects. The study was approved by the ethics committee of Hannover Medical School (Nr. 5498). Additionally, this study was performed in accordance to the ethical standards laid down in the 1964 Declaration of Helsinki. This study was carried out from 2010 to 2017.

### 2.1. Patients

Thirty-two voluntary female FMS patients aged from 18 to 70 years were recruited from outpatients of the Department of Rehabilitation Medicine of Hannover Medical School and the patient organisation “RheumaLiga”, Hannover. Patients were screened based on the definition of the American College of Rheumatology (ACR) criteria for fibromyalgia [25] by physicians of the Department of Rehabilitation Medicine, Hannover Medical School, Hannover, Germany. Additionally, patients had to understand the German language. They were not restricted with regard to treatments, either pharmacological or non-pharmacological. However, the treatments were recorded and the patients were obliged to stop any food and medicine intake one night before blood collection.

Patients with recent or past history of psychiatric disorders (e.g., major depressive disorder, alcohol dependence, substance abuse, schizophrenic or paranoid disorder, personality disorders, and somatoform disorders), inflammatory, endocrine or clinically significant chronic disease (e.g., diabetes, rheumatoid arthritis, inflammatory bowel disease, and organic brain disorders), abnormal function of liver, pregnant and breastfeeding women were excluded.

### 2.2. Healthy Control (HC)

The range of age defined in the inclusion criteria of the study for HCs was 18–70 years old. Thirteen female HCs in the range of 44–68 years old were recruited and selected. Exclusion criteria were, in particular, chronic pain, pregnancy and breastfeeding women.

### 2.3. Assessment Tools

Except illness duration (years), which was only for FMS patients, the following variables were also evaluated in HCs: pain intensity (Visual Analogue Scales (VAS)), and intensity of fatigue (VAS), as well as depression (Hospital anxiety and depression score [HADS]-D) and anxiety (HADS-A) [26].

### 2.4. Identification of Monocytes and MK2 Analysis Methods

**Figure 1:**
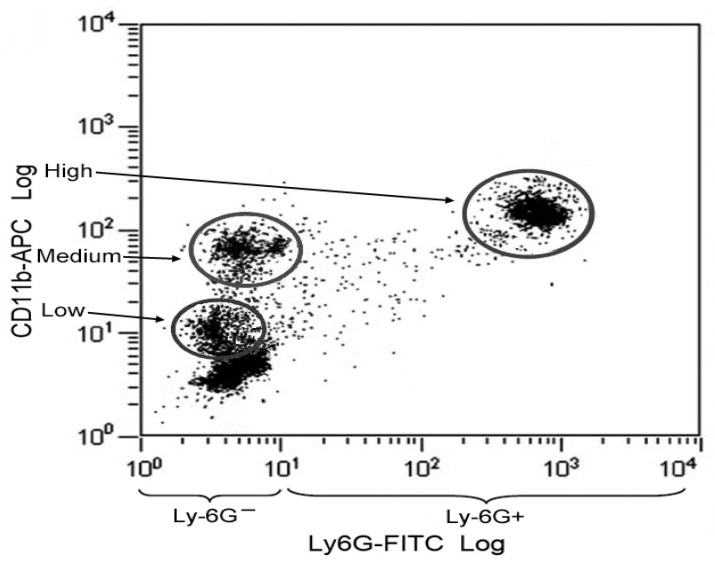
Monocytes identification from whole blood cells. NK cells: CD11b^Low^ and Ly-6G^−^ cells; Monocytes: CD11b^Medium^ and Ly-6G^−^ cells (Zhao et al., 2008). Neutrophils: CD11b^High^ Ly-6G^+^ cells (Comalada et al., 2003).

After overnight fasting (only water was allowed), peripheral venous blood samples were collected in anticoagulant (K-EDTA) tubes from all patients and controls between 8:00 and 10:00 a.m. MK2 was analyzed according to the method described by Zhao et al. [24]. Briefly, whole blood was collected in K-EDTA tubes (Sarstedt^®^, Nümbrecht, Germany). FITC-conjugated anti-Ly-6G (1:250; RB6-8C5, ab25024; Abcam Biochemicals, Cambridge, UK) and APC-conjugated anti-CD11b (1:100; BD Biosciences, Becton Dickinson GmbH, Heidelberg, Germany) were added into 100 μL of blood and incubated at 37 °C for 15 min. To lyse and remove red blood cells and fix white blood cells, 20 times volume of Lyse/Fix buffer was added and vortexed. The cells were then collected by centrifugation and washed twice with staining/washing buffer. According to the in vivo cell-based assay that was initially developed to identify specific p38 MAPK inhibitors [24], we investigated the activation of the p38 MAPK pathway in monocytes of whole blood from HCs and FMS patients. First, we stained the whole human blood cells with antibodies to Ly-6G and CD11b to separate neutrophils from the other myeloid cells and lymphocytes. Based on positive CD11b cell surface staining, three distinct CD11b staining-positive populations were observed: CD11bHigh, CD11bMedium, and CD11bLow. As reported previously [24,27], the CD11bHigh cells showed positive antibody staining for Ly-6G, a specific cell surface antigen for neutrophils [27]. Both CD11bMedium and CD11bLow subsets were negative for Ly-6G. According to Zhao et al. [24], those cells were identified as nonlymphoid cells, such as blood monocytes and NK cells. The CD11bMedium and Ly-6G− cells have been further identified previously as monocytes, while the CD11bLow and Ly-6G− cells were NK cells (Zhao et al. 2008). Thus, the subsets of blood myeloid cell can be identified based on CD11b-positive staining. As Figure 1 shows, this method led to a distinct differentiation of determined cells.

For the intracellular staining of Phospho-MAPKAPK-2, cells were resuspended in 200 μL of permeabilization buffer with anti-Phospho MAPKAPK-2 (Thr334; Cell Signaling, New England Biolabs GmbH, Frankfurt, Germany), incubated at room temperature for 30 min, collected by centrifugation, and washed twice by the staining/washing buffer (phosphate-buffered saline with 0.09% NaN3 and 5% fetal bovine serum). Cells were resuspended in 200 μL of staining/washing buffer with anti-immunoglobulin-PE conjugate (1:250; BD Biosciences, Becton Dickinson GmbH, Heidelberg, Germany) and incubated at room temperature for 30 min. The cells were collected by centrifugation, and washed twice with staining/washing buffer. Finally the stained cells were collected and analyzed by flow cytometry (Becton Dickinson, Franklin Lakes, NJ, USA).

### 2.5. Calculation of the Area of Phosphorylation of MK2

**Figure 2:**
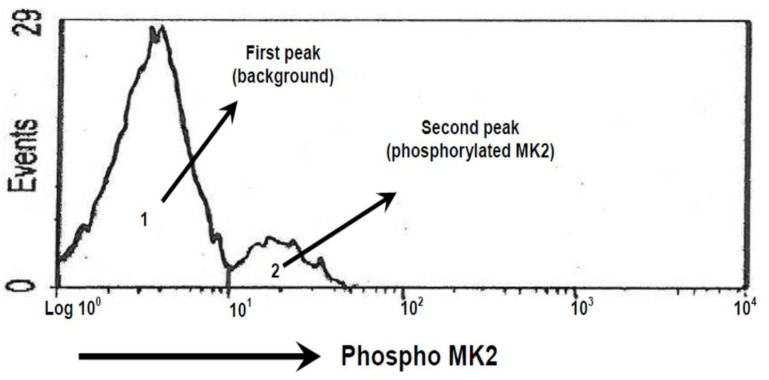
Area of Phosphorylated MK2.

The peak of MK2 activity was measured as area under curve. This was digitized and calculated by using UN-SCAN-IT software (Silk Scientific Corporation, Orem, UT, USA). Area of phosphorylated MK2 was defined as a second peak with cut-off at log 101 (Figure 2).

### 2.6. Statistics Analysis

Shapiro-Wilk test was used to check normality of the data. Based on these results, all clinical and demographic parameters as well as area of phosphorylated MK2 were tested using Student’s *t*-test to compare FMS patients and HCs. Subgroup analysis: Student’s *t*-test was used to compare the level of area phosphorylated of MK2 in FMS patients with and without antidepressant drug intake. Significance of the results was set as *p* < 0.05. Statistic package SPSS 25.0 was used.

## 3. Results

### 3.1. Characteristics of FMS Patients and HCs

The age of FMS patients and HCS was not significantly different (Table 1). However, as expected, the other clinical characteristics, such as pain, fatigue, anxiety, and depression scores, were significantly higher in FMS patients as compared to HC.

### 3.2. MK2 Measurement

**Figure 3:**
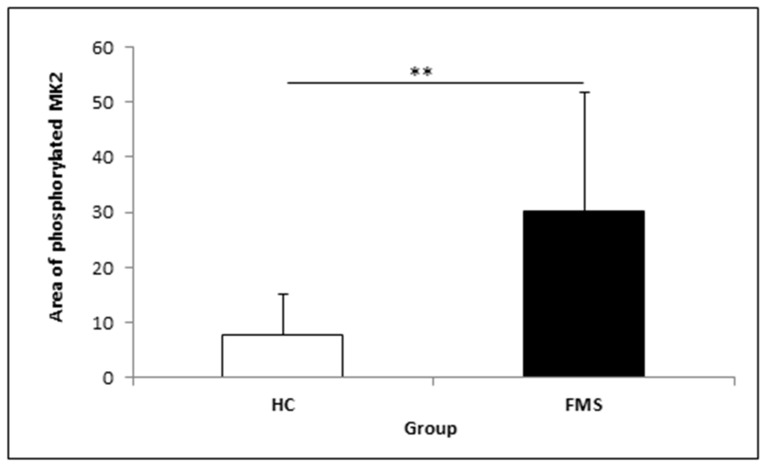
Area of phosphorylated MK2 in peripheral blood monocytes of HCs and FMS patients. (** Student’s *t*-test: *p* < 0.001).

Figure S1 (Supplement) shows an example of the event counts of the measurements of MK2 activity of HCs and FMS patients. All FMS patients show a peak related to phosphorylated MK2 that is not clearly seen in HCs. The statistical analysis of the area of the activated MK2 showed a significantly higher level of MK2 in FMS patients as compared to HCs (Figure 3). The mean value of activated MK2 of FMS patients is about fivefold higher as compared to HCs (FMS: 49.99 ± 4.51; HCs: 9.89 ± 2.01).

**Figure 4:**
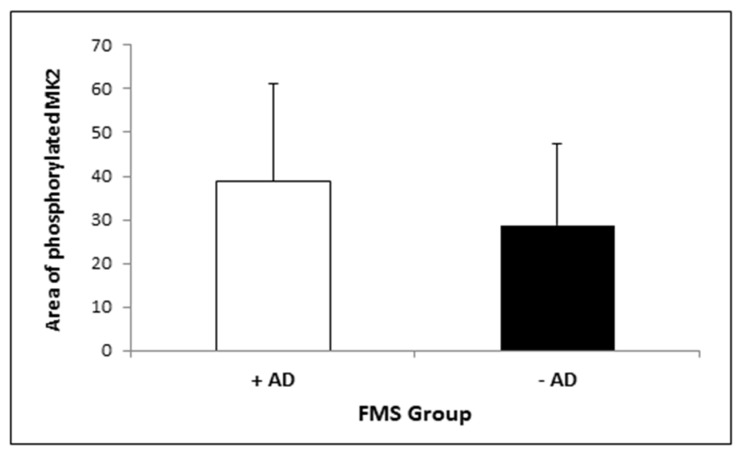
Area of phosphorylated MK2 in peripheral blood monocytes of FMS patients with antidepressant (+AD) and without antidepressant (−AD) intake. (Data are presented in Mean (SD); Student’s *t*-test: *p* = 0.094).

To analyse possible effects of antidepressant intake, a subgroup analysis was performed in FMS patients only (Figure 4). The figure demonstrates the tendency of lower MK2 activity in FMS patients who took antidepressants; however, it was not significantly different from FMS patients without antidepressant intake.

## 4. Discussion

One of the aims of this study was to determine MK2 activity in monocytes (defined as CD11bMediumLy-6G− cells) [24] of whole blood samples in FMS patients and HCs. The result of our study showed markedly higher levels of activated MK2 in monocytes of FMS patients as compared to those of HCs. This shows that the p38 MAPK-MK2 pathway is activated in monocytes of FMS patients.

Zhao et al. [24] demonstrated in mouse whole blood cells that only monocytes, but not NK cells or nonlymphoid cells, expressed high levels of p38α kinase, which could be rapidly activated in vitro by treatment with anisomycin. Anisomycin is a known synthetic stimulator of p38 MAPK-MK2 signaling and rapidly activates this pathway [27,28]. Here, we determined MK2 activity in monocytes (CD11bMedium and Ly-6G− cells) in FMS patients as compared to HC. The results showed that this method was successfully adapted for human sample, too.

Circulating monocytes are a major source of pro-inflammatory cytokine production, and the p38 MAPK-MK2 pathway is known to determine the posttranscriptional regulation of cytokine biosynthesis [23,29] and account for inflammation (Tilton et al. 2006). Additionally, the p38 MAPK-MK2 pathway is essential in controlling the production of cytokines in response to various stressors [12]. Therefore, monocytes are of particular interest for studying p38 MAPK signaling.

Although many studies reported that FMS is not an inflammatory pain syndrome, some reports suggested FMS to be related to inflammatory disorder, as they found increased levels of different pro-inflammatory cytokines in FMS [30]. This observation is supported by recent results of meta-analysis demonstrating elevated levels of IL-6 in FMS patients as compared to healthy subjects [31]. However, the results are not conclusive due to conflicting data in the literature [31,32]. The authors suggested that further studies with a larger sample size are still needed to confirm this hypothesis. Our findings may support the hypothesis that inflammatory mechanisms are involved in pathophysiology of FMS.

On the other hand, the p38 MAPK pathway is linked to the development of both inflammatory and neuropathic pain. Our observation is of interest in several ways, as it has been demonstrated previously that the p38 MAPK pathway is related to pain and depression [33,34], which are symptoms that are often observed in FMS patients.

Furthermore, we investigated a possible link of activated p38 MAPK-MK2 signaling and depression in FMS patients. Depression as a long-term consequence of stress can be linked to altered GR function as well as MAPK activities in animal models [35]. In addition, the p38 MAPK is known to inhibit GR function, and changes in p38 MAPK levels by depression may modulate the glucocorticoid action. In line with the observed depression and dysregulation of glucocorticoid levels in FMS patients [5], p38 MAPK activity may reflect a part of the pathomechanism implicated in the development of the FMS [36]. This activity is mediated by MK2 [12]. However, in this study, a clear correlation between MK2 activity and depression in FMS patients could not be observed. This result may be due to the fact that we excluded patients with MDD and those with depressive symptoms have been treated with antidepressant drugs. Another reason for the different results could be due to the different disease models. FMS is a chronic pain syndrome with mood-related disorders as comorbidity, while a chronic stress model was used in the previous study [35].

The hypothesis of autonomic nervous system dysfunction in the aetiology of FMS patients has been emerging since the last two decades [37]. It is supported by clinical and experimental studies, such as anomaly response to various stimuli (e.g., auditory stimulation, cold pressor tests) and the nocturnal heart rate variability in FMS that correlated with pain severity and depression [37,38,39,40]. In FMS patients, as aforementioned, the immune system is dysregulated, particularly related to NK subset cells family [41,42]. It has been known that the autonomic nervous system interacts with the immune system [43]. This interaction is also mediated by the p38/MAPK pathway [43]. Therefore, p38/MAPK could also play a role in autonomic nervous system dysfunction in FMS patients.

There is a relevance of muscle pathology in understanding FMS, disturbance of cytokine networks between psychologic stressors of FMS and infection/inflammation [36,44]. The activation of the p38 MAPK pathway as a critical mediator can lead to central pain sensitization in FMS patients. The fact that MAPK pathway participates in the generation and maintenance of inflammatory pain [45] may lead to development of inhibitor of this pathway for pain management. This study reports the activation of the p38 MAPK pathway in FMS patients.

Limitations. This study showed interesting results which demonstrate higher activity of the MK2 pathway in FMS patients. However, we had limitations in this study. Patients were not asked to stop the drug intake for a certain period of time in order to wash out the drug effect, especially antidepressants. This was decided to avoid ethical issues and difficulties to predict washout period of different types of drugs. However, in this study, antidepressants did not seem to influence the activity of MK2. Another study should be performed with a larger sample size and considering other confounding factors. Another limitation is that we could not do sample size calculation due to the fact that was an explorative study and no similar study has been performed before.

Future direction. For FMS patients, a multimodal approach consisting of a combination of pharmacology and non-pharmacology interventions has been recommended [46]. Exercise is one of the recommended non-pharmacology interventions which can improve chronic pain and induce changes in mood [46,47]. In addition, regular endurance aerobic exercise also leads to changes in active p38 MAPK and MK2 levels. Widegren et al. (2001) reported that, in the initial exercise phase, the phosphorylated p38 MAPK and MK2 is induced but that activity of p38 MAPK signaling is reduced after weeks of aerobic exercise in trained as compared to untrained healthy subjects [48]. In addition, this training program alters the levels of inflammatory cytokines, such as TNF- α or IL-6, to induce overall beneficial health effects [49]. Since exercise is one of the recommended treatments for FMS patients, this kinase pathway may be relevant for a number of beneficial effects achieved by exercise in chronic pain syndromes like FMS. Finally further studies are needed to elucidate the relationship between activated MK2 as determined in monocytes and exercise in the treatment of FMS patients.

## 5. Conclusions

Our data demonstrate that the human blood cell-based intracellular MK2 phosphorylation as an assay to determine activated MK2 in monocytes of a selected group of FMS patients is an appropriate method. Based on the combination of CD11b, Ly-6G, and MK2 antibody staining with flow cytometry method, we could efficiently identify these monocytes in whole blood cells. Our result suggests that activity of the p38/MK2 axis can be a clinical marker for FMS. In addition, the p38 MAPK pathway may be involved in the pathomechanism of FMS. It may be relevant for the future development of novel treatment strategies in FMS, either pharmacological or non-pharmacological.

## Figures and Tables

**Table 1:** Clinical characteristics of HCs and FMS patients. Data (Mean ± SD).

	HC (*n* = 13)	FMS (*n* = 32)	*p*
Age (years)	52.77 ± 6.50	54.59 ± 9.10	0.456
Pain Intensity (VAS)	0.70 ± 0.52	5.75 ± 1.95	<0.001
Fatigue (VAS)	1.58 ± 2.14	5.83 ± 2.46	<0.001
Anxiety (HADS-A Score)	4.69 ± 2.92	9.08 ± 4.28	0.002
Depression (HADS-D Score)	2.54 ± 2.54	7.48 ± 4.28	<0.001

Note: HCs: Healthy controls; FMS: fibromyalgia syndrome; VAS: Visual Analogue Scale; HADS-A: Hospital Anxiety and Depression Score-Anxiety; HADS-D: Hospital Anxeity and Depression Score-Depression; Significant: Student’s *t*-test.

## Data Availability

The datasets used and/or analyzed during the present study are available from the corresponding author on reasonable request.

## Supplementary Materials

The following are available online at https://www.mdpi.com/article/10.3390/medicina57040396/s1, Figure S1: Examples of MK2 activation in blood monocytes of HC and FMS patients.

## References

[1] Sawaddiruk P., Paiboonworachat S., Chattipakorn N., Chattipakorn S.C. (2017). Alterations of brain activity in fibromyalgia patients. J. Clin. Neurosci..

[2] Staud R. (2002). Evidence of involvement of central neural mechanisms in generating fibromyalgia pain. Curr. Rheumatol. Rep..

[3] Staud R., Smitherman M.L. (2002). Peripheral and central sensitization in fibromyalgia: Pathogenetic role. Curr. Pain Headache Rep..

[4] Casale R., Sarzi-Puttini P., Botto R., Alciati A., Batticciotto A., Marotto D., Torta R. (2019). Fibromyalgia and the concept of resilience. Clin. Exp. Rheumatol..

[5] Schoneveld O.C.J., Cidlowski J.A. (2007). Glucocorticoids and immunity: Mechanisms of regulation. Psychoneuroimmunology.

[6] Macedo J.A., Hesse J., Turner J.D., Ammerlaan W., Gierens A., Hellhammer D.H., Muller C.P. (2007). Adhesion molecules and cytokine expression in fibromyalgia patients: Increased L-selectin on monocytes and neutrophils. J. Neuroimmunol..

[7] Santarelli S., Zimmermann C., Kalideris G., Lesuis S.L., Arloth J., Uribe A., Dournes C., Balsevich G., Hartmann J., Masana M. (2017). An adverse early life environment can enhance stress resilience in adulthood. Psychoneuroendocrinology.

[8] Tsigos C., Kyrou I., Kassi E., Chrousos G.P., Feingold K.R., Anawalt B., Boyce A., Chrousos G., de Herder W.W., Dungan K., Grossman A., Hershman J.M., Hofland J., Kaltsas G. (2000). Stress: Endocrine Physiology and Pathophysiology. Endotext.

[9] Miller A.H., Haroon E., Raison C.L., Felger J.C. (2013). Cytokine targets in the brain: Impact on neurotransmitters and neurocircuits. Depress. Anxiety.

[10] Altier N., Stewart J. (1998). Dopamine receptor antagonists in the nucleus accumbens attenuate analgesia induced by ventral tegmental area substance P or morphine and by nucleus accumbens amphetamine. J. Pharm. Exp. Ther..

[11] Coulthard L.R., White D.E., Jones D.L., McDermott M.F., Burchill S.A. (2009). p38(MAPK): Stress responses from molecular mechanisms to therapeutics. Trends Mol. Med..

[12] Gaestel M. (2006). MAPKAP kinases—MKs—Two’s company, three’s a crowd. Nat. Rev. Mol. Cell Biol..

[13] Ji R.R., Suter M.R. (2007). p38 MAPK, microglial signaling, and neuropathic pain. Mol. Pain.

[14] Yang G., Chang C.C., Yang Y., Yuan L., Xu L., Ho C.T., Li S. (2018). Resveratrol Alleviates Rheumatoid Arthritis via Reducing ROS and Inflammation, Inhibiting MAPK Signaling Pathways, and Suppressing Angiogenesis. J. Agric. Food Chem..

[15] Zhang T., Zhang N., Zhang R., Zhao W., Chen Y., Wang Z., Xu B., Zhang M., Shi X., Zhang Q. (2018). Preemptive intrathecal administration of endomorphins relieves inflammatory pain in male mice via inhibition of p38 MAPK signaling and regulation of inflammatory cytokines. J. Neuroinflamm..

[16] Anand P., Shenoy R., Palmer J.E., Baines A.J., Lai R.Y., Robertson J., Bird N., Ostenfeld T., Chizh B.A. (2011). Clinical trial of the p38 MAP kinase inhibitor dilmapimod in neuropathic pain following nerve injury. Eur. J. Pain.

[17] Auffray C., Sieweke M.H., Geissmann F. (2009). Blood monocytes: Development, heterogeneity, and relationship with dendritic cells. Annu. Rev. Immunol..

[18] Italiani P., Boraschi D. (2014). From Monocytes to M1/M2 Macrophages: Phenotypical vs. Functional Differentiation. Front. Immunol..

[19] Pereira M., Petretto E., Gordon S., Bassett J.H.D., Williams G.R., Behmoaras J. (2018). Common signalling pathways in macrophage and osteoclast multinucleation. J. Cell Sci..

[20] Grage-Griebenow E., Flad H.D., Ernst M. (2001). Heterogeneity of human peripheral blood monocyte subsets. J. Leukoc Biol..

[21] Tilton J.C., Johnson A.J., Luskin M.R., Manion M.M., Yang J., Adelsberger J.W., Lempicki R.A., Hallahan C.W., McLaughlin M., Mican J.M. (2006). Diminished production of monocyte proinflammatory cytokines during human immunodeficiency virus viremia is mediated by type I interferons. J. Virol..

[22] Han J., Lee J.D., Tobias P.S., Ulevitch R.J. (1993). Endotoxin induces rapid protein tyrosine phosphorylation in 70Z/3 cells expressing CD14. J. Biol. Chem..

[23] Neininger A., Kontoyiannis D., Kotlyarov A., Winzen R., Eckert R., Volk H.D., Holtmann H., Kollias G., Gaestel M. (2002). MK2 targets AU-rich elements and regulates biosynthesis of tumor necrosis factor and interleukin-6 independently at different post-transcriptional levels. J. Biol. Chem..

[24] Zhao J., Evans G., Li W., Green L., Chu S., Marder P., Na S. (2008). Rapid and quantitative detection of p38 kinase pathway in mouse blood monocyte. Vitr. Cell Dev. Biol. Anim..

[25] Wolfe F., Smythe H.A., Yunus M.B., Bennett R.M., Bombardier C., Goldenberg D.L., Tugwell P., Campbell S.M., Abeles M., Clark P. (1990). The American College of Rheumatology 1990 Criteria for the Classification of Fibromyalgia. Report of the Multicenter Criteria Committee. Arthritis Rheum..

[26] Zigmond A.S., Snaith R.P. (1983). The hospital anxiety and depression scale. Acta Psychiatry Scand..

[27] Comalada M., Xaus J., Valledor A.F., Lopez-Lopez C., Pennington D.J., Celada A. (2003). PKC epsilon is involved in JNK activation that mediates LPS-induced TNF-alpha, which induces apoptosis in macrophages. Am. J. Physiol. Cell Physiol..

[28] Andersson K., Sundler R. (2000). Signalling to translational activation of tumour necrosis factor-alpha expression in human THP-1 cells. Cytokine.

[29] Tiedje C., Ronkina N., Tehrani M., Dhamija S., Laass K., Holtmann H., Kotlyarov A., Gaestel M. (2012). The p38/MK2-driven exchange between tristetraprolin and HuR regulates AU-rich element-dependent translation. PLoS Genet..

[30] Ortega E., Bote M.E., Giraldo E., Garcia J.J. (2012). Aquatic exercise improves the monocyte pro- and anti-inflammatory cytokine production balance in fibromyalgia patients. Scand. J. Med. Sci. Sports.

[31] Uceyler N., Hauser W., Sommer C. (2011). Systematic review with meta-analysis: Cytokines in fibromyalgia syndrome. BMC Musculoskelet. Disord..

[32] Nugraha B., Korallus C., Gutenbrunner C. (2013). Serum level of brain-derived neurotrophic factor in fibromyalgia syndrome correlates with depression but not anxiety. Neurochem. Int..

[33] Yasuda S., Sugiura H., Tanaka H., Takigami S., Yamagata K. (2011). p38 MAP kinase inhibitors as potential therapeutic drugs for neural diseases. Cent. Nerv. Syst. Agents Med. Chem..

[34] Humo M., Ayazgok B., Becker L.J., Waltisperger E., Rantamaki T., Yalcin I. (2020). Ketamine induces rapid and sustained antidepressant-like effects in chronic pain induced depression: Role of MAPK signaling pathway. Prog. Neuro-Psychopharmacol. Biol. Psychiatry.

[35] Budziszewska B., Szymanska M., Leskiewicz M., Basta-Kaim A., Jaworska-Feil L., Kubera M., Jantas D., Lason W. (2010). The decrease in JNK- and p38-MAP kinase activity is accompanied by the enhancement of PP2A phosphate level in the brain of prenatally stressed rats. J. Physiol. Pharm..

[36] Bennett R. (2005). Fibromyalgia: Present to future. Curr. Rheumatol. Rep..

[37] Buskila D. (2009). Developments in the scientific and clinical understanding of fibromyalgia. Arthritis Res. Ther.

[38] Staud R. (2008). Heart rate variability as a biomarker of fibromyalgia syndrome. Future Rheumatol..

[39] Raj S.R., Brouillard D., Simpson C.S., Hopman W.M., Abdollah H. (2000). Dysautonomia among patients with fibromyalgia: A noninvasive assessment. J. Rheumatol..

[40] Qiao Z.G., Vaeroy H., Morkrid L. (1991). Electrodermal and microcirculatory activity in patients with fibromyalgia during baseline, acoustic stimulation and cold pressor tests. J. Rheumatol..

[41] Nugraha B., Korallus C., Kielstein H., Gutenbrunner C. (2013). CD3+CD56+natural killer T cells in fibromyalgia syndrome patients: Association with the intensity of depression. Clin. Exp. Rheumatol..

[42] Dimmek J.D., Korallus C., Buyny S., Gutenbrunner C., Lichtinghagen R., Jacobs R., Nugraha B. (2021). Brain-Derived Neurotrophic Factor and Immune Cells in Osteoarthritis, Chronic Low Back Pain, and Chronic Widespread Pain Patients: Association with Anxiety and Depression. Medicina.

[43] Kenney M.J., Ganta C.K. (2014). Autonomic nervous system and immune system interactions. Compr. Physiol..

[44] Albrecht P.J., Rice F.L. (2016). Fibromyalgia syndrome pathology and environmental influences on afflictions with medically unexplained symptoms. Rev. Environ. Health.

[45] Mai L., Zhu X., Huang F., He H., Fan W. (2020). p38 mitogen-activated protein kinase and pain. Life Sci..

[46] Hauser W., Ablin J., Perrot S., Fitzcharles M.A. (2017). Management of fibromyalgia: Practical guides from recent evidence-based guidelines. Pol. Arch. Intern. Med..

[47] Macfarlane G.J., Kronisch C., Dean L.E., Atzeni F., Hauser W., Fluss E., Choy E., Kosek E., Amris K., Branco J. (2017). EULAR revised recommendations for the management of fibromyalgia. Ann. Rheum. Dis..

[48] Widegren U., Ryder J.W., Zierath J.R. (2001). Mitogen-activated protein kinase signal transduction in skeletal muscle: Effects of exercise and muscle contraction. Acta Physiol. Scand..

[49] Handschin C., Spiegelman B.M. (2008). The role of exercise and PGC1alpha in inflammation and chronic disease. Nature.

