# Supplementary figures and images for: The p38/MK2 Axis in Monocytes of Fibromyalgia Syndrome Patients: An Explorative Study
Source: Medicina (Kaunas). 2021 Apr 19;57(4):396. doi: 10.3390/medicina57040396 (PMC8072914; doi:10.3390/medicina57040396)

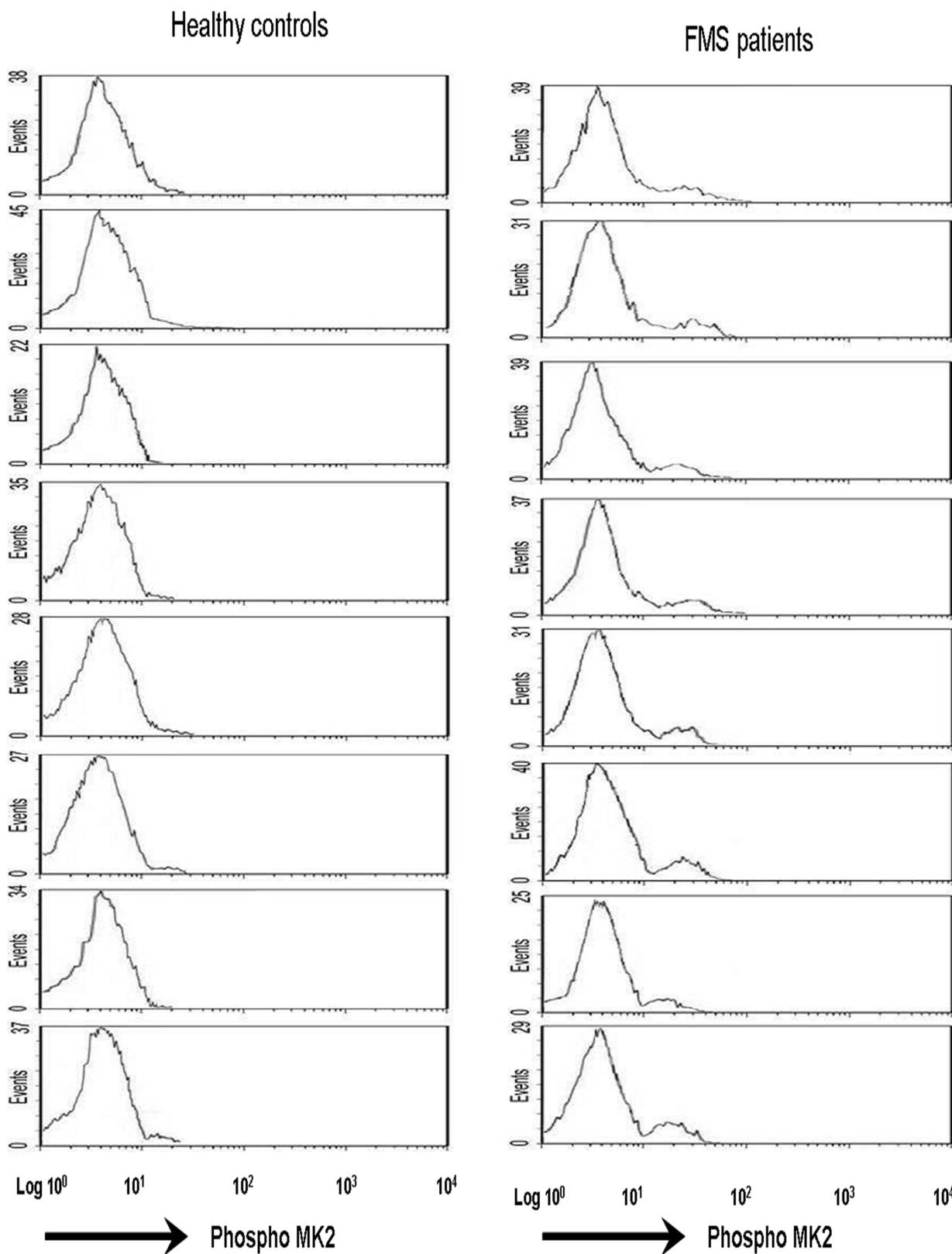

Figure S1 Examples of MK2 activation in blood monocytes of HC and FMS patients

Supplement: Supplementary file 1 [file medicina-57-00396-s001.zip › medicina-1147977-supplementary.pdf]
